# Intrinsic and extrinsic factors influencing the clinical course of B-cell chronic lymphocytic leukemia: prognostic markers with pathogenetic relevance

**DOI:** 10.1186/1479-5876-7-76

**Published:** 2009-08-28

**Authors:** Michele Dal-Bo, Francesco Bertoni, Francesco Forconi, Antonella Zucchetto, Riccardo Bomben, Roberto Marasca, Silvia Deaglio, Luca Laurenti, Dimitar G Efremov, Gianluca Gaidano, Giovanni Del Poeta, Valter Gattei

**Affiliations:** 1 Clinical and Experimental Onco-Hematology Unit, Centro di Riferimento Oncologico, I.R.C.C.S., Aviano (PN), Italy; 2 Laboratory of Experimental Oncology and Lymphoma Unit, Oncology Institute of Southern Switzerland, Bellinzona, Switzerland; 3 Division of Hematology and Transplant, Department of Clinical Medicine and Immunological Sciences, University of Siena, Siena, Italy; 4 Division of Hematology – Department of Oncology and Hematology-University of Modena and Reggio Emilia, Modena, Italy; 5 Laboratory of Immunogenetics, Department of Genetics, Biology and Biochemistry and CeRMS, University of Turin, Turin, Italy; 6 Hematology Institute, Catholic University "Sacro Cuore", Rome, Italy; 7 Molecular Hematology, ICGEB Outstation-Monterotondo, Rome, Italy; 8 Division of Hematology – Department of Clinical and Experimental Medicine & BRMA – Amedeo Avogadro University of Eastern Piedmont, Novara, Italy; 9 Chair of Hematology, S.Eugenio Hospital and University of Tor Vergata, Rome, Italy

## Abstract

B-cell chronic lymphocytic leukemia (CLL), the most frequent leukemia in the Western world, is characterized by extremely variable clinical courses with survivals ranging from 1 to more than 15 years. The pathogenetic factors playing a key role in defining the biological features of CLL cells, hence eventually influencing the clinical aggressiveness of the disease, are here divided into "intrinsic factors", mainly genomic alterations of CLL cells, and "extrinsic factors", responsible for direct microenvironmental interactions of CLL cells; the latter group includes interactions of CLL cells occurring via the surface B cell receptor (BCR) and dependent to specific molecular features of the BCR itself and/or to the presence of the BCR-associated molecule ZAP-70, or via other non-BCR-dependent interactions, e.g. specific receptor/ligand interactions, such as CD38/CD31 or CD49d/VCAM-1. A putative final model, discussing the pathogenesis and the clinicobiological features of CLL in relationship of these factors, is also provided.

## Introduction

B-cell chronic lymphocytic leukemia (CLL) is a monoclonal expansion of small mature B lymphocytes accumulating in blood, marrow, and lymphoid organs. Despite a remarkable phenotypic homogeneity, CLL is characterized by extremely variable clinical courses with survivals ranging from one to more than 15 years [1]. In this regard, specific chromosomal aberrations (i.e. 17p-, 11q- or +12), as well as the presence of an unmutated (UM) rather than mutated (M) status of immunoglobulin (IG) heavy chain variable (*IGHV*) genes, or expression levels for ZAP-70, CD38 and CD49d exceeding the value of an established threshold, have been reported to correlate with a poor clinical outcome in CLL [2-8].

In the present review, the main factors playing a role in defining the biological features of CLL cells, hence eventually influencing the clinical aggressiveness of the disease, are divided into "intrinsic factors", mainly genomic alterations of CLL cells, and "extrinsic factors", responsible for direct micro-environmental interactions of CLL cells.

### Intrinsic factors

Under the terms "intrinsic factors" are gathered the major genomic alterations associated with a CLL phenotype. Such alterations can be either primarily responsible for the first step(s) of neoplastic transformation of B cells (primary genetic lesions, e.g. 13q14.3 deletion, see below) or acquired during disease progression, also as a consequence of microenvironmental interactions (i.e. secondary genetic lesions). Telomer lenght too was included in this chapter, although often consequence of environmental factors affecting cell proliferation (see below).

It is common notion that, differently from other B-cell lymphoid neoplasms, CLL is characterized by recurrent DNA gains and losses and not by the presence of specific chromosomal translocations. However, using either improved protocols to obtain informative metaphases [9,10] or microarray-based comparative genomic hybridization [11], chromosomal abnormalities can now be detected in over 90% of patients [9]. Only a fraction of the events are balanced translocations, whilst the vast majority of them are unbalanced translocations (see below), determining losses or gains of genomic material [9,10]. Specific genomic events are associated with a different clinical outcome and, the frequency of specific genomic events varies between CLL bearing Mutated (M) and Unmutated (UM) *IGHV *genes (see below for *IGHV *molecular features). The recurrent chromosomal aberrations are summarized in Table 1.

**Table 1:** Intrinsic factors with prognostic relevance

**Karyotype**	**% of cases, range**^**a**^	**Prognosis **	**Known and/or putative involved genes**
**13q14.3 loss**	14–40	good^b^	*mir-16-1*; *mir-15a*

**11q22-23 loss**	10–32	bad	*ATM*

**trisomy 12**	11–18	intermediate	*CLLU1*

**17p13.1 loss**	3–27	bad	*TP53*

^a^According to [30];

^b^If the sole genetic aberration.

#### 13q14.3 deletion

The most common lesion in CLL is chromosome 13q14.3 deletion, occurring in half of the cases [4]. The deletion is often interstitial and can be homozygous in up to 15% of the cases [4]. When it represents the only lesion it is associated with a good clinical outcome, and with the presence of Mutated *IGHV *genes [4,10,12]. A selective advantage, possibly proning B cell clones to additional mutations, could be conferred because of the high frequency of 13q deletion [13].

The pathogenetic role of 13q deletion in CLL is not fully clear, although its high frequency has suggested a primary and central role in the CLL transformation process [14]. Several regions between 130 and 550 kb were described, all comprising a minimal deleted region of 29 kb located between exons 2 and 5 of *DLEU2 *[15]. The deleted region always comprises the locus coding for two microRNAs (miRNAs), hsa-mir-16-1 and hsa-mir-15a [15], but it can also include the region coding for the retinoblastoma gene (RB1) [16]. *mir-16-1 *and *mir-15a *are deleted or downregulated in the majority (about 70%) of CLL [14]. miRNAs represent a large class of regulating non-coding small RNA molecules, acting by binding messenger RNAs and determining their degradation or inhibition of translation [17]. Over-expression of the anti-apoptotic *BCL2*, due to the reduced negative regulation by *mir-16-1 *and *mir-15a*, has been proposed along with other several genes often involved in cell cycle and/or programmed cell death regulation such as *MCL1*, *ETS1 *and *JUN *[16,18-20]. Additional studies are needed to identify the genes actually involved in CLL pathogenesis via the 13q deletion.

#### Trisomy 12

The trisomy 12 bears an intermediate prognosis and is only marginally associated with an UM *IGHV *gene status [10,12]. The 12q22 segment contains *CLLU1 *which is the first gene that was considered specific for CLL cells, but no difference in *CLLU1 *protein expression in patients with or without trisomy 12 has been reported [21,22]. Of note, high *CLLU1 *expression levels has been demonstrated to predict poor clinical outcome in CLL of younger patients [23].

#### 11q22-q23 deletion

CLL harboring 11q22-q23 deletion tend to present a rapidly evolving disease [4]. This lesion targets the gene coding for *ATM *(ataxia telangiectasia mutated), which is mutated in approximately 15% of CLL, not necessarily bearing concomitant 11q losses [24]. The presence of 11q deletion or of *ATM *mutations determines poor prognosis, and it is more common among cases with UM *IGHV *and ZAP-70 or CD38 positivity, or experiencing bulky lymphadenopathies [4,10,24-28]. ATM is involved in the DNA repair and its inactivation impairs the response of CLL cells to chemotherapy [26,28]. It has been suggested that, for the complete lack of *ATM *function, the other *ATM *allele should present mutations [29]. Since *ATM *mutations are present in one third of the 11q- cases, the poor prognosis of 11q- patients has been suggested to depend on mechanisms involving other genes affecting cell cycle regulation and apoptosis (e.g. *NPAT*, *CUL5*, *PPP2R1B*) [28,29].

#### 17p13.1 deletion

The recurrent 17p13.1 deletion, affecting TP53, occurs only in a small fraction of CLL patients at diagnosis [4]. It confers the worst prognosis among all the genetic lesions [4], and it is more common among patients bearing other poor prognostic factors, such as UM *IGHV*, or ZAP-70 and CD38 expression [4,10,27,30]. TP53 is a transcription factor activated by strand breaks in DNA that is involved in triggering cell apoptosis and/or cell-cycle arrest, with the aim to maintain the genome integrity by hindering clonal progression [31]. The activation of *TP53 *is tightly regulated by the *MDM2 *(murine double minute-2) gene [32], whose expression is regulated in part by a TP53 responsive promoter. MDM2, an E3 ubiquitin ligase for TP53 and itself, controls TP53 half-life via ubiquitin-dependent degradation [33-35]. In cells with functional TP53, the TP53 activity is primarily inhibited through direct and tonic interaction with the MDM2 protein [32]. Treatment of various tumor cells with inhibitors of the MDM2-TP53 interaction results in rising TP53 levels and subsequent induction of cell cycle arrest and apoptosis [36]. Thus, small-molecule inhibitors that block the MDM2-TP53 interaction, like Nutlins, could represent a new therapeutic strategy for treatment of CLL patients [37].

In CLL, *TP53 *is mutated in about 10% of patients at presentation and in 10% to 30% of patients with pretreated disease [38-40]. *TP53 *can be inactivated by somatic mutations which can occur in the presence or in the absence of any genomic loss [2,25]. Whereas up to two-thirds of del17p13 CLL also harbor *TP53 *mutations, a fraction of CLL carries *TP53 *mutations without del17p13 [2,25,41], and *TP53 *mutations have been shown to have a negative prognostic relevance also in the absence of *TP53 *deletion [42]. Besides *TP53 *mutations and deletion, other mechanisms of TP53 dysfunction may be operative in CLL [28,43-46]. These mechanisms may involve the *ATM *and *MDM2 *genes that regulate TP53 function at the protein level [28,46]. ATM is related to TP53 because it acts as a TP53 kinase, although *ATM *deletions do not confer a disease as aggressive as it occurs in *TP53 *deletions [47]. Notably, *ATM *mutations and *MDM2 *polymorphisms causing aberrant MDM2 expression have been shown to harbor prognostic relevance in CLL [28,43,46].

TP53 inactivation is associated with a poor response to chemotherapy, including alkylating agents and purine analogues [2]. This suggested the need, for patients affected by CLL with disrupted TP53 function, of TP53 independent therapeutic agents [26,41,48,49]. In this regard, CLL that at diagnosis presented del17p13 without *TP53 *mutations displayed a significantly longer time to chemorefractoriness than CLL with *TP53 *mutations already at diagnosis [42]. In addition, CLL with del17p13 only acquired *TP53 *mutations at chemorefractoriness [42].

#### Chromosomal translocations and other chromosomal abnormalities

Historically, chromosomal translocations were considered infrequent events in CLL. However, relatively recent studies reported an unexpected high frequency (approximately 20%) of reciprocal translocations when successful methods for CLL B cell stimulation are employed, e.g. by utilizing CD40 ligand or oligonucleotides and IL-2 as stimuli [9,50]. These studies have also correlated chromosomal translocations with shorter treatment-free survival and overall survival. Together with the more common chromosomal abnormalities, genome wide screening has found other alterations consisting of clonal monoallelic and biallelic losses as well as gains such as duplications, amplifications and trisomies [51-54]. These alterations concern relatively small chromosomal regions spread throughout the CLL genome [51-54]. Moreover, these gains or losses enable the detection of clonal variants that differ at several loci [52]. The biologic and prognostic significance of these other recurrent genomic aberrations is not known. Patients bearing three or more aberrations or chromosomal translocations might have a worse prognosis [9]. Prospective trials and a more widespread use of genome wide techniques to assess CLL genome will help to identify further genetic prognostic markers.

#### Telomere length

An interesting feature of CLL is its heterogeneity in terms of telomere length and telomerase (hTERT) activity [55-58]. Short telomeres and high hTERT activity are associated with worse clinical outcome, with an UM *IGHV *gene status, with high ZAP-70, CD38, and CD49d expression, as well as with specific cytogenetic abnormalities [56,58,59]. Regarding this latter point, short telomeres are frequently associated with 11q or 17p deletions whereas long telomeres are present in 13q- patients [58]. Normal B cells in the germinal center present high hTERT activity, and telomere elongation has been shown to occur at the same time of the somatic hypermutation process [60], thus, B cells with M *IGHV *genes present longer telomeres than B cells with UM genes. Therefore it is conceivable that different B cells already present different telomere length before the leukemic transformation; alternatively, kinetic characteristics of CLL cells can determine differences in telomere length, and telomere shortening might be a consequence of 11q- or 17p- aberration that, together with ZAP-70, CD38 and CD49d overexpression, results in a more rapid CLL cell turnover, facilitating survival and cell-cycle progression [58,61].

#### Clinical implications of intrinsic factors

In the clinical practice, the detection, by using a panel of interphase fluorescence in situ hybridization (FISH) probes, at least including 13q14.3, 11q22-23 and 17p13.1 deletions and trisomy 12, should always be part of the initial diagnostic procedure. Although only a small portion of patients presents genetic abnormalities considered bad prognostic markers, such as 17p or 11q deletions, at the onset, these alterations can appear during the clinical course, more often in patients carrying other poor prognostic markers (such as UM *IGHV *mutational status or high ZAP-70, CD38 and CD49d expression) [38,39]. Given that acquisition of new cytogenetic abnormalities may influence the response to therapy, FISH analysis should be repeated at the time of progression or before therapy selection. Given its valuable prognostic impact, analysis of *TP53 *mutational status could be also advisable in the phase of progressive disease.

### Extrinsic factors

Extrinsic factors are responsible for direct interaction of CLL cells with other micro-environmental cell populations. In the present review, we focused on interactions of CLL cells occurring via the surface B cell receptor (BCR) and dependent on specific molecular features of the BCR itself and/or on the presence of the BCR-associated molecule ZAP-70, or via other non-BCR-dependent interactions, e.g. the CD38/CD31 or CD49d/VCAM-1 receptor/ligand interactions (Table 2). Differences in *IGHV *mutational status and in BCR functionality suggested a different cell of origin for CLL with UM versus CLL with M *IGHV *gene mutational status. Despite this, CLL cases appear very homogenous when their gene expression profiles are compared with those of normal or other neoplastic B-cells [62,63]. For this reason CLL is nowadays believed to derive from subsets of marginal zone memory B-cells that have undergone either a T-cell dependent or T-cell independent maturation [64,65].

**Table 2:** Extrinsic factors with prognostic relevance

**Factors**	**Negative prognosis if expressing**	**Cases with unfavourable values, mean % (range)**	**Putative mechanisms responsible for unfavourable prognosis**
**BCR**	- *UM IGHV* - stereotyped BCR? - *M IGHV3-23*?	42.3 (40–46)^a^	- high reactivity or polyreactivity - superantigens recognition?

**ZAP-70 **	>20%	44.7 (36–52)^b^	- tyrosine phosphorylation - calcium influx - chemokine sensitivity

**CD38 **	>30%	36.3 (30–44)^c^	- microenviromental interactions (CD38/CD31)

**CD49d **	>30%	36.5 (28–43)^d^	- microenviromental interactions (CD49d/VCAM-1; CD49d/Fibronectin)

^a^Deduced from [25,91,92];

^b^Deduced from [7,114,116];

^c^Deduced from [25,127,128];

^d^Deduced from [8,150,152].

#### The BCR in CLL

BCR is a multimeric complex constituted of a membrane-bound IG glycoprotein and a heterodimer IGα/IGβ (CD79A/CD79B), located on the surface of B cell. The IG glycoprotein is composed by two identical heavy chains (μ, δ, α, γ or ε) and two identical light chains: κ or λ. Both heavy and light chains have two variable regions (*IGHV *or *IG*(*K*/*L*)*V*) that mediates antigen contact and vary extensively between IG, along with a constant region that is responsible for the effector activities. For heavy chain, the variable region is encoded by three gene segments: variable (*IGHV*), diversity (*IGHD*) and joining (*IGHJ*), whereas the variable regions of the light chains are generated from *IG(K/L)V *and *IG(K/L)J *segments. Both for heavy and light chains, the segments involved in *V(D)J *recombination confer diversity by random and imprecise rearrangement during B-cell development in the bone marrow. The consequent protein sequences mainly differ in the complementary-determining-region-3 of the heavy (HCDR3) and light (K/LCDR3) chains. Diversity is further enhanced by the somatic hypermutation (SHM) process, which requires BCR cross-linking by the antigen, cellular activation, cooperation of T lymphocytes and other cells, and introduces point mutations in variable regions of rearranged immunoglobulin heavy and light chains [66]. Another process physiologically occurring during B cell differentiation is the so-called class-switch recombination (CSR), which modify the constant region of heavy chains, thus altering the effector functions of IG [66].

The BCR has always been a key molecule to understanding CLL, initially only due to the surface IG that were utilized to make or support a correct diagnosis [67]. Surface IG are usually IGM/IGD, expressed at low/dim intensity [47]. The explanation of the low/dim expression level of BCR is still unclear [47]. CLL expressing IGG is a relatively rare variant whose origin and antigenic relation with the most common IGM/IGD variant is still not completely clear [68].

Studies of the molecular structure of the BCR in CLL are suggesting evidences of a promoting role of the antigen encounter. A first evidence has been provided by analysis of *IGHV *genes starting in the early 90s' that revealed that 50% of CLL had M *IGHV *genes [69-71]. These mutations often fulfill the criteria for selection by antigen with more replacement mutations in heavy chain complementarity determining regions (HCDR) and less in heavy chain framework regions (HFR), which permits the development of a more specific antigen-binding site by maintaining the necessary supporting scaffold of BCR [6,72-76].

From a clinical point of view, in 1999, two mutually confirmatory papers demonstrated that somatic mutations correlated with more benign diseases. In fact, a CLL subgroup with very unfavourable clinical outcome presents none or few (<2%) mutations (UM CLL) in *IGHV *genes, respect to the closest germ line sequence. CLL cells of this particular subgroup seem to receive continuous anti-apoptotic and/or proliferating microenvironmental stimuli via BCR leading to a more aggressive disease than the subgroup with M configuration of *IGHV *genes (≥2%; M CLL), respect to the closest germ line sequence [3,77]. A difference in outcome was also demonstrated in patients receiving an autologous stem-cell transplant (ASCT); all patients with UM *IGHV *genes undergoing ASCT relapsed and progressed after a 4-year follow-up, while most with M *IGHV *genes remained in molecular remission at this stage [78].

Activation-induced cytidine deaminase (AID), an enzyme involved in SHM and CSR during normal B cell differentiation [79], was found to be upregulated in UM CLL cells [80], and, even if expression could be restricted to a small fraction of the clone [6,81], AID seems to be functional with generation of isotype-switched transcripts and mutations in the pre-switch μ region [82,83]. AID upregulation causes mutation in genes related with an aggressive disease (e.g. *BCL6*, *PAX5*, *MYC*, *RHOH*) [84,85]. Furthermore, a relation of AID expression with deletions in 11q- and loss of TP53 has been found [86].

#### BCR stereotypes in CLL (see Figure 1)

**Figure 1:**
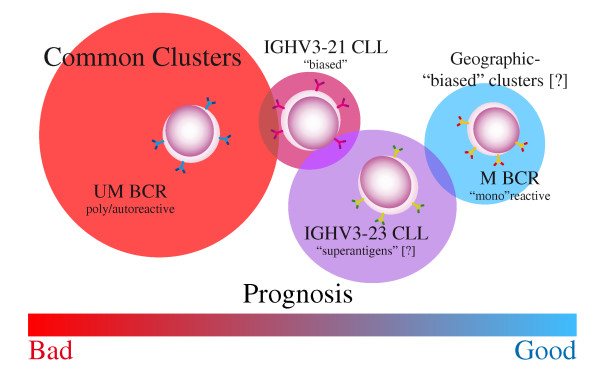
**CLL subsets with distinct BCR features and their correlation with prognosis**. Question marks in parenthesis indicate data that has to be confirmed by further investigations.

CLL have a biased use of some specific gene segments. For example, a preferential use of *IGHV1-69 *in the UM CLL and *IGHV4-34 *and *IGHV3-23 *in the M CLL subgroups has been documented [87-92]. In addition, several groups reported the existence of subsets of CLL cases carrying BCR characterized by non-random pairing of specific *IGHV*, highly homologous or identical HCDR3 often associated with a restricted selection of *IGVK *or *IGVL *light chains (the so-called "stereotyped BCR") [89-97]. These stereotyped BCR have been detected in more than 20% of CLL cases [89,91,92,97]; the non-random composition of the expressed BCR on the CLL cells with IG binding lead to hypothesize a specificity for similar/identical antigens [89,91,92,98].

The chance of carrying a stereotyped BCR is higher for UM CLL [94]. The vast majority of the clusters shared by distinct, and in several cases geographically distant, datasets ("common" clusters) were composed by UM cases [89,91-94,97]. In particular, these UM clusters included cases that seem to express both autoreactive and polyreactive BCR, allegedly deriving from the B cell compartment devoted to the production of natural antibodies [96,98,99]. Among "common" clusters, of particular clinical interest is a cluster composed by UM CLL with stereotyped BCR expressing genes from the *IGHV1 *gene family other than *IGHV1-69 *(*IGHV1-2*,*IGHV1-18*, *IGHV1-3*,*IGHV1-46*, *IGHV7-4-1*), homologous HCDR3 bearing the QWL amino acid motif, and *IGKV1-39 *light chains [89,91,92]. The prognosis of CLL expressing this stereotyped BCR is poor either if compared to all the other patients affected by M or UM CLL, or only to the cases expressing the same *IGHV *genes but without the same stereotyped BCR [89,92].

Among the few M clusters that are shared by the majority/totality of the datasets, there are two clusters, both expressing IGG, composed by cases expressing *IGHV4-34 *and *IGHV4-39*, respectively [89,91,92,100,101]. Specific cluster-biased genomic aberrations have been found; 13q- has been associated with *IGHV4-34/IGKV2-30 *cluster while trisomy 12 has been associated with the *IGHV4-39/IGKV1-39 *cluster [101]. Interestingly, the latter cluster has been associated with the development of Richter syndrome in CLL [102,103]. Other clusters, mainly composed by M cases and expressing *IGHV3 *subgroup genes, are less frequent and might be subjected to a geographical bias.

Finally, of particular interest is a group of *IGHV3-21 *CLL, composed by cases with either UM or M *IGHV *genes, that expressed a stereotyped BCR characterized by an unusually short and highly homologous HCDR3 associated with *IGLV3-21 *[88,90,93,104,105]. Of note, a significantly skewed representation of this particular cluster has been well documented in different European and non-European countries and even in different regions from the same country [88,90,104,105]. From a clinical standpoint, evidence is provided that patients belonging to *IGHV3-21/IGLV3-21 *CLL cluster have shorter TTT when compared to all M CLL and to M CLL expressing *IGHV3-21 *but not included in this stereotyped cluster [88,90,92]. Although the issue is still controversial [88,105], the molecular basis for a more aggressive clinical behaviour of CLL belonging to *IGHV3-21/IGLV3-21 *CLL cluster is also suggested by gene expression profiling and immunophenotypic analyses [90]. The notion that only patients affected by CLL belonged to the *IGHV3-21/IGLV3-21 *cluster experience a more progressive disease may have important implications given the proposal of using *IGHV3-21 *expression to drive clinical decision in prospective trials [27,49].

#### Non-stereotyped BCR in CLL (see Figure 1)

Considering the *IGHV *gene usage and relating it with the distribution of *IGHV *gene in stereotyped BCR clusters, it has been observed that cases expressing the *IGHV3-23 *gene are constantly absent from stereotyped BCR clusters [106], despite that *IGHV3-23 *is the second most frequently used and usually M *IGHV *gene in CLL [89,90,92]. A possible explanation justifying the absence of *IGHV3-23 *genes from clusters of stereotyped BCR is the possibility that *IGHV3-23*-expressing BCR might be selected through non-CDR-based recognition mechanisms, e.g. through interactions with superantigens, a general feature of BCR expressing *IGHV3 *subgroup genes [106-109]. From a clinical standpoint, hints suggesting a negative prognostic impact of *IGHV3-23 *usage in CLL have been reported [110]. Recently, such a suggestion has been confirmed in an Italian multicenter series, but circumscribed to cases expressing mutated *IGHV *genes [106]. In this series, median TTT of M *IGHV3-23 *patients were significantly shorter than median TTT of M non-*IGHV3-23 *CLL, and *IGHV3-23 *expression was identified as an independent negative prognosticator in the context of M CLL [106].

#### ZAP-70

ZAP-70 encodes for T cell specific zeta-associated protein-70 and has been initially identified in T cells as a protein tyrosine kinase that plays a critical role in T-cell-receptor signaling [111]. This molecule is a member of the syk family of tyrosine kinases and is associated with the ζ-chain of the CD3 complex [112].

Gene expression profiling studies in CLL, aimed at identifying differentially expressed genes between UM and M CLL, described ZAP-70 as the most differentially expressed gene between the two CLL subtypes, thus highlighting a high correlation between ZAP-70 expression and IGHV mutational status [63,113]. Consistently, ZAP-70 was shown to act as surrogate for *IGHV *gene mutations when its intra-cytoplasmic expression is investigated by flow cytometry [5,7,114-116], although a common standardized protocol for its detection is still to be defined [7,114,115,117]. However, discordance of ZAP-70 expression and *IGHV *mutational status was reported in about 25% of cases with a higher number of discordant cases in subgroups with a more aggressive disease such as 11q- CLL, 17p- CLL or *IGHV3-21 *CLL (39%) [118]. Using a cut-off set at 20% of positive cells, ZAP-70 expression was demonstrated to have a negative prognostic impact in CLL [5,7]. The relevance of ZAP-70 as independent prognosticator was provided by multivariate analysis [116].

ZAP-70 can modulate BCR-derived signaling associating with BCR in antigen stimulated CLL cells [119], and can play an indirect role in BCR signal transduction, mainly modulating events at the end of the signaling response [120]. Expression of ZAP-70, which can enhance and prolong on syk and other downstream signaling molecules, can partially determine the different capability of CLL cells to respond to antigenic stimulation [120]. Regarding the mechanism(s) underlying the negative prognostic impact of ZAP-70 expression in CLL, it is known that ZAP-70^+ ^CLL cells have a greater capacity to respond to antigen-induced signals through BCR triggering. In particular, ZAP-70 expression and sustained BCR stimuli have been associated with prolonged activation of the Akt and ERK kinases, events which are required for the induction of several antiapoptotic proteins, including Mcl-1, Bcl-xL and XIAP [120-122]. Recently, ZAP-70 expression was demonstrated to mark CLL subsets with enhance capability to respond to chemokine-mediated stimuli (see below).

#### CD38

CD38 is a 45-kDa type II membrane glycoprotein first described as an activation antigen whose expression coincided with discrete stages of human T and B lymphocyte differentiation [123]. CD38 has been found to be widely expressed in humans within the hematopoietic system (e.g. bone marrow progenitor cells, monocytes, platelets and erytrocytes) and beyond, in brain, prostate, kidney, gut, heart and skeletal muscle [124]. CD38 behaves simultaneously as a cell surface enzyme and as a receptor. As an ectoenzyme, CD38 synthesizes cyclic adenosine diphosphate (ADP) ribose and nicotinic acid adenine dinucleotide phosphate (NAADP), key compounds in the regulation of cytoplasmic Ca^++ ^levels [125]. Engagement of CD38 by its ligand CD31 or by specific agonist antibodies induces activation and differentiation signals in T, B and NK cells [126]. Signals mediated by CD38 are tightly regulated by the dynamic localization of the molecule in lipid microdomains within the plasma membrane, and by lateral associations with other proteins or protein complexes [124].

A study by Damle et al. indicated that CD38 expression was heterogeneous among CLL cases [3]. By using a given percentage of CLL cells expressing the antigen (30% of positive cells), significant prognostic differences were found by investigating both chemotherapy requirements and overall survival [3]. The same report showed that CLL cells with higher CD38 expression more likely rearranged UM *IGHV *genes [3]. Thus, CD38 status was proposed as surrogate for *IGHV *mutation status, although this was not confirmed by subsequent studies, which however substantiated the its independent prognostic significance [12,127-131].

These observations on the prognostic relevance of CD38 found a biologic ground in studies indicating that CLL cell growth and survival were favoured through sequential interactions between CD38 and CD31 and between CD100 and plexin B1, the latter expressed by microenvironmental cells [132,133]. These interactions are more likely to occur in peripheral lymphoid organs and/or bone marrow given the higher CD38 expression in residential as opposed to circulating CLL cells [134-136]. Moreover, both bone marrow and peripheral lymphoid organs can provide accessibility to CD31, as endothelial, stromal, and the so-called nurse-like cells all express high-CD31 levels [137-139]. Necessary condition for CD38-mediated signals are CD38 translocation into lipid rafts and lateral association with CD19, which is also part of the so-called "tetraspan web" (CD19/CD81), and comprises different molecules, including β1 integrins such as CD49d [140]. Moreover, CD38^+ ^CLL cells, expecially if coexpressing ZAP-70, are characterized by enhanced migration toward CXCL21/SDF-1α, and CD38 ligation leads to phosphorylation of the activatory tyrosines in ZAP-70 [133,141]. Therefore, ZAP-70 represents a cross-point molecule where migratory signals mediated via the CXCL21 receptor CXCR4 intersect with growth signals mediated via CD38 [142-144]. Finally, the associated expression of CD38 and CD49d (see below) can provide additional mechanisms explaining the poor prognosis of CD38-expressing CLL.

#### CD49d

CD49d, a.k.a. α4 integrin, acts primarily as an adhesion molecule capable of mediating both cell-to-cell interactions, via binding to vascular-cell adhesion molecule-1 (VCAM-1), and interactions with extracellular matrix components by binding to non-RGD sites (a.k.a. CS-1 fragments) of fibronectin (FN), as well as the C1q-like domain of elastin microfibril interfacer-1 (Emilin-1) [145,146]. In this regard, CD49d-expressing CLL cells were shown to have a high propensity to adhere to fibronectin substrates, and an increased CD49d protein expression was demonstrated in CLL cells from advanced Rai stage patients [147]. Our group recently collected evidences of VCAM-1 over-expression in the stromal-endothelial component found in the context of lymphoid aggregates in bone marrow biopsies (BMB) of CD49d/CD38-expressing CLL [148]. VCAM-1 upregulation was demonstrated to be due to an overproduction by CD38/CD49d-expressing CLL cells of specific chemokines (CCL3 and CCL4) upon CD38 triggering, eventually capable to recruit TNFα-producing macrophages, which in turn are responsible for VCAM-1 upregulation by stromal/endothelial cells [148]. VCAM-1/CD49d interactions resulted in an increased survival of CD49d-expressing CLL cells [148]. CD49d-dependent interactions have a role in preventing both spontaneous and drug induced apoptosis of normal or neoplastic B cells [145,149]. Moreover, chemokine-induced transmigration of CLL cells across endothelia depends on CD49d expression by CLL cells and is favoured by the production of the matrix metalloproteinase-9 as the result of CD49d engagement [150].

From a clinical point of view, CD49d has been identified as an independent negative prognosticator for CLL, marking a subset of CLL patients characterized by aggressive and accelerated clinical course [8,150-152]. The prognostic relevance of CD49d in CLL may have also therapeutic implications, envisioning the use for CLL patients of Natalizumab (TYSABRI, Biogen Idec, Cambridge, MA and Elan Pharmaceuticals, South San Francisco, CA, USA), a humanized anti-CD49d monoclonal antibody already available and currently employed in autoimmune diseases such as multiple sclerosis and Crohn's disease [153].

## Conclusion (see Figure 2)

**Figure 2:**
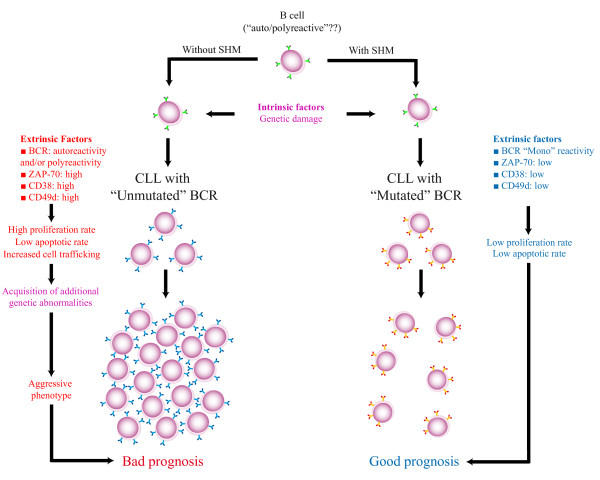
**A "multistep" model for CLL origin**.

B cells carrying BCR with high affinity for autoantigens are usually deleted or addressed towards a secondary rearrangement of heavy/light chains; in the latter case, B cells that reach an "acceptable" ("non-autoreactive") structure are then driven to continue differentiation [154,155]. In some istances, such secondary attempts may fail and B cell clones may retain an "inappropriate" reactivity (autoreactivity, polyreactivity) [156]. As an example, many normal B cell clones with UM *IGHV *genes produce antibodies capable of a certain degree of polyreactivity by binding multiple antigens (e.g. carbohydrates, nucleic acids, phospholypids). If one of these cells presents or develops primary genetic abnormalities (e.g. 13q14.3 deletions, but also other lesions) it can undergo leukemic transformation. B cells with genetic abnormalities and UM/polyreactive BCR can increase their number through repeated expositions to antigens (foreign antigens, autoantigens) [71,157]. In this regard, immune cross-reactivity between exogenous polysaccharide/carbohydrate antigens and autoantigens is not infrequent [158,159]. Together with BCR, other factors, usually highly expressed in UM CLL, such as ZAP-70, CD38 and CD49d might take part in strengthening the "proliferative" and/or "pro-survival" interactions of CLL cells with microenvironment [122,133,148,160]. Such a "proliferative" status also allows CLL cells to acquire additional/secondary genetic changes, transforming them into a more aggressive phenotype [13].

Moreover, the expression of high levels of surface molecules, such as CD38 and CD49d, may facilitate the trafficking of CLL cells in the context of bone marrow and/or lymph nodes where interactions with microenvironmental cells marked by "nurse-like" activities are easier to occur [132,137-139,148]. In this regard, it has been hypothesized that the highest proliferation rate occurs mainly/exclusively in the context of a tiny proportion of tumor cells (i.e. the so-called "tumor initiating cells" a.k.a. "cancer stem cells"), frequently clustered to form sort of pseudofollicolar proliferation centers in lymph nodes and bone marrow [139], but also present in peripheral blood as "circulating cancer stem cells" with features of "side population" in flow cytometry cytograms after fluorescent vital dye staining [161].

Similar mechanism(s) might be hypothesized for M CLL. Also in this case, intrinsic and extrinsic factors may take part in the neoplastic transformation but unlike UM CLL, in M CLL the BCR might be selected by a sole antigen (autoantigen or foreign antigen) or by a group of antigens with very similar characteristics, often with evidence of a geographic-biased distribution [92,105]. This "mono-reactivity" might determine a less aggressive pathology [3,6,77]. Of note, somatic hypermutation of *IGV *genes can decrease autoreactivity levels [99]. It is possible to hypothesize that given the less aggressive clinical course, in some cases CLL cells of a mutated clone may be anergic, with an attenuated response to BCR engagement [162-164]. The low expression of CD38 and CD49d, usually associated with a M *IGHV *gene status in CLL, fails to provide additional microenvironmental stimuli.

The hypothesis of a "multistep" origin for CLL is in keeping studies describing the presence of B cells with CLL cell features in about 3.5% of healthy people, allegedly representing a clonal amplification of a selected set of B lymphocytes [165,166].

## Competing interests

The authors declare that they have no competing interests.

## Authors' contributions

MDB wrote the manuscript, FB, FF, AZ, RB, RM, SD, LL, DGE, GG, GDP contributed to write the manuscript and VG contributed to write and revised the manuscript.
